# Genome Sequences of Clinical *Vibrio cholerae* Isolates from an Oyster-Borne Cholera Outbreak in Florida

**DOI:** 10.1128/genomeA.00966-13

**Published:** 2013-11-21

**Authors:** Bradd J. Haley, Seon Young Choi, Nur A. Hasan, Abdul Shakur H. Abdullah, Thomas A. Cebula, Anwar Huq, Rita R. Colwell

**Affiliations:** Maryland Pathogen Research Institute, University of Maryland, College Park, Maryland, USAa; CosmosID, College Park, Maryland, USAb; Department of Biology, Johns Hopkins University, Baltimore, Maryland, USAc; Maryland Institute for Applied Environmental Health, School of Public Health, University of Maryland, College Park, Maryland, USAd; University of Maryland Institute for Advanced Computer Studies, University of Maryland, College Park, Maryland, USAe; Johns Hopkins University Bloomberg School of Public Health, Baltimore, Maryland, USAf

## Abstract

Between November 2010 and April 2011, 11 cases of cholera were identified and associated with the consumption of raw oysters harvested from Apalachicola Bay, Florida. The etiological agent was the *ctxAB*-positive *Vibrio cholerae* serogroup O75. The genome sequences of the isolates provide useful information and are deposited in the public genome databases.

## GENOME ANNOUNCEMENT

*Vibrio cholerae*, the causative agent of cholera, a gastrointestinal infection causing profuse rice water diarrhea, is known to be autochthonous to aquatic environments worldwide. Currently, >200 *V. cholerae* serogroups have been identified, with the majority of cholera cases being ascribed to *V. cholerae* serogroups O1 and O139. However, it is now known that mobile and genome-anchored virulence factors are responsible for the characteristic symptoms of cholera, regardless of serogroup. Further, recent large-scale molecular analyses of *V. cholerae* isolates recovered from cholera outbreaks demonstrated that a significant number of cases of cholera are caused by infection with *V. cholerae* non-O1 or non-O139 serogroups (1–3). Thus, it is imperative to focus attention on strains other than those of the O1 and O139 serogroups to describe more completely the public health burden of *V. cholerae* on a global scale. In an effort to accomplish this, the genomes of eight *V. cholerae* serogroup O75 isolates from a single cholera outbreak were sequenced.

Between November 2010 and April 2011, 11 cases of cholera were reported in four states (Florida [8 cases], Georgia, Louisiana, and Indiana [1 case each]) that were concurrent with, but genetically unrelated to, the cholera epidemic that started in Haiti in November 2010 (4). The majority of patients reported having eaten raw oysters harvested from the Apalachicola Bay, Florida, prior to the onset of symptoms. The clinical isolates were identified as *V. cholerae* serogroup O75 and found to contain the cholera toxin genes (*ctxAB*), but no further molecular analyses were conducted. Interestingly, *V. cholerae* O75 has been isolated repeatedly, albeit sporadically, from both clinical cholera cases and the environment in the southeastern United States (5).

Using the Illumina genome analyzer IIx system (Illumina, Inc., San Diego, CA) according to the manufacturer’s methods, the genomes of *V. cholerae* O75 strains CP1110, CP1111, CP1112, CP1113, CP1114, CP1115, CP1116, and CP1117 were sequenced. Genomic DNA was extracted using the Qiagen DNeasy blood and tissue kit (Qiagen, Valencia, CA). Coverage was between 196× and 348×, and raw reads were assembled using the CLC Genomics Workbench, generating between 183 and 207 contigs. The genomes were annotated using the RAST server (6). The estimated sizes of the *V. cholerae* O75 genomes ranged between 3,925,419 and 3,932,707 bp (3,479 and 3,489 protein coding sequences). A detailed report of the results of comparative analyses of these genomes and other available *V. cholerae* genomes will be published elsewhere.

### Nucleotide sequence accession numbers.

The sequences for the *V. cholerae* O75 strains were deposited at NCBI under accession no. AMWF00000000 (CP1110), AMWS00000000 (CP1111), AMWT00000000 (CP1112), AMWU00000000 (CP1113), AMWV00000000 (CP1114), AMWR00000000 (CP1115), ANNM00000000 (CP1116), and AMWW00000000 (CP1117).
